# No functional TRPA1 in cardiomyocytes

**DOI:** 10.1111/apha.13659

**Published:** 2021-05-04

**Authors:** Clara Hoebart, Natalia S. Rojas‐Galvan, Cosmin I. Ciotu, Ibrahim Aykac, Lukas F. Reissig, Wolfgang J. Weninger, Attila Kiss, Bruno K. Podesser, Michael J. M. Fischer, Stefan Heber

**Affiliations:** ^1^ Center for Physiology and Pharmacology Medical University of Vienna Vienna Austria; ^2^ Center for Biomedical Research Medical University of Vienna Vienna Austria; ^3^ Division of Anatomy Medical University of Vienna Vienna Austria

**Keywords:** cardiomyocytes, heart, human, mouse, TRPA1, TRPV1

## Abstract

**Aim:**

There is mounting evidence that TRPA1 has a role in cardiac physiology and pathophysiology. We aim to clarify the site of TRPA1 expression in the heart and in particular whether the channel is expressed in cardiomyocytes.

**Methods:**

Due to the high calcium conductance of TRPA1, and marginal calcium changes being detectable, microfluorimetry in primary mouse cardiomyocytes, and in the cardiomyocyte cell lines H9c2 and HL‐1, was applied. TRPA1 mRNA in mouse and human hearts, primary cardiomyocytes, and the cardiac cell lines were quantified. Dorsal root ganglia served as control for both methods.

**Results:**

In addition to AITC, the more potent and specific TRPA1 agonists JT010 and PF‐4840154 failed to elicit a TRPA1‐mediated response in native and electrically paced primary cardiomyocytes, and the cardiomyocyte cell lines H9c2 and HL‐1. There were only marginal levels of TRPA1 mRNA in cardiomyocytes and cardiac cell lines, also in conditions of cell differentiation or inflammation, which might occur in pathophysiological conditions. Similarly, TRPV1 agonist capsaicin did not activate primary mouse cardiomyocytes, did not alter electrically paced activity in these, and did not activate H9c2 cells or alter spontaneous activity of HL‐1 cells. Human pluripotent stem cells differentiated to cardiomyocytes had no relevant TRPA1 mRNA levels. Also in human post‐mortem heart samples, TRPA1 mRNA levels were substantially lower compared with the respective dorsal root ganglion.

**Conclusion:**

The results do not question a role of TRPA1 in the heart but exclude a direct effect in cardiomyocytes.

## 1 INTRODUCTION

Transient receptor potential channel subtype ankyrin 1 (TRPA1) is an ion channel activated by a multitude of electrophilic substances and thus serves as a sensor for potentially noxious molecules.^1^, ^2^ Upon activation, the channel opens and causes a depolarization of the TRPA1‐expressing cell by allowing cations to enter, with a particularly high permeability for calcium ions. TRPA1 is expressed by nociceptive primary sensory neurons, which are a subset of the peptidergic neurons in the dorsal root ganglion (DRG) and trigeminal ganglion (TG),^3^, ^4^ where it plays an important role in pain perception.^5^

A selective TRPA1 activation is sufficient to elicit pain in humans,^6^ and the absence of the channel in mice leads to a great reduction in nocifensive behaviour when exposed to environmental irritants.^7^, ^8^ Therefore, TRPA1 has been subject to extensive research as an attractive drug target to treat pain, but so far targeting TRPA1 has not translated into clinical options.^9^

However, neuronal and nonneuronal expression^10^ allow also to assume a nonanalgesic potential of TRPA1 inhibition, for example, modifying pathophysiology and disease progression.^11^ Several studies have reported TRPA1 expression, to a lesser extent, in various other cell types,^12^, ^13^, ^14^, ^15^, ^16^ including cardiomyocytes. In murine cardiomyocytes, TRPA1 expression has been reported at mRNA,^17^, ^18^, ^19^ protein,^20^, ^21^ and functional level.^22^ In addition, a regulation of its expression in cardiac tissue by insulin‐like growth factor has been described.^18^ Most important is whether TRPA1 contributes to cardiac physiology and pathophysiology. To understand the respective mechanisms, it appears essential to know whether and on which cells functional TRPA1 can be found in the heart. A functional expression in cardiomyocytes would have rendered cardiac side effects likely when targeting TRPA1. As evidence is mounting that TRPA1 modulation could have a role in cardiac pathophysiology, the contribution of TRPA1 in the different cell types needs to be addressed.

Application of TRPA1 agonist allyl isothiocyanate (AITC) was reported to increase cardiomyocyte contractility by enhancing calcium transients in a Ca^2^⁺/calmodulin‐dependent protein kinase II‐dependent manner.^20^ There is emerging evidence that TRPA1 might also play a key role in cardiac pathophysiology.^23^, ^24^ Nonetheless, its contribution in the context of myocardial ischemia remains ambiguous, because TRPA1 activation was reported to be detrimental^19^ but also to be protective.^17^

Recently, it was reported that TRPA1 and transient receptor potential vanilloid 1 (TRPV1), two functionally and potentially even structurally interlinked receptor channels,^25^, ^26^, ^27^ are co‐expressed in cardiac tissue. This was shown by western blots, immunohistochemistry, and functional assays, using AITC and capsaicin as agonists.^21^ However, in a transgenic reporter mouse model for TRPV1, the ion channel was not expressed in cardiomyocytes, but in nerve endings.^28^ Given that there has been discrepancy regarding the expression pattern within the myocardium, the aim of this study was to clarify the presence of functional TRPA1 and TRPV1 in cardiomyocytes. To this end, we quantified TRPA1 and TRPV1 mRNA in commonly used cardiomyocyte and myoblast cell lines, in primary cardiomyocytes from mice, and in human myocardium. In addition, more specific pharmacological tools than in previous publications were used to evaluate TRPA1 and TRPV1 function.

## 2 RESULTS

### 2.1 No evidence for functional expression of TRPA1 or TRPV1 in primary murine cardiomyocytes

**FIGURE 1:**
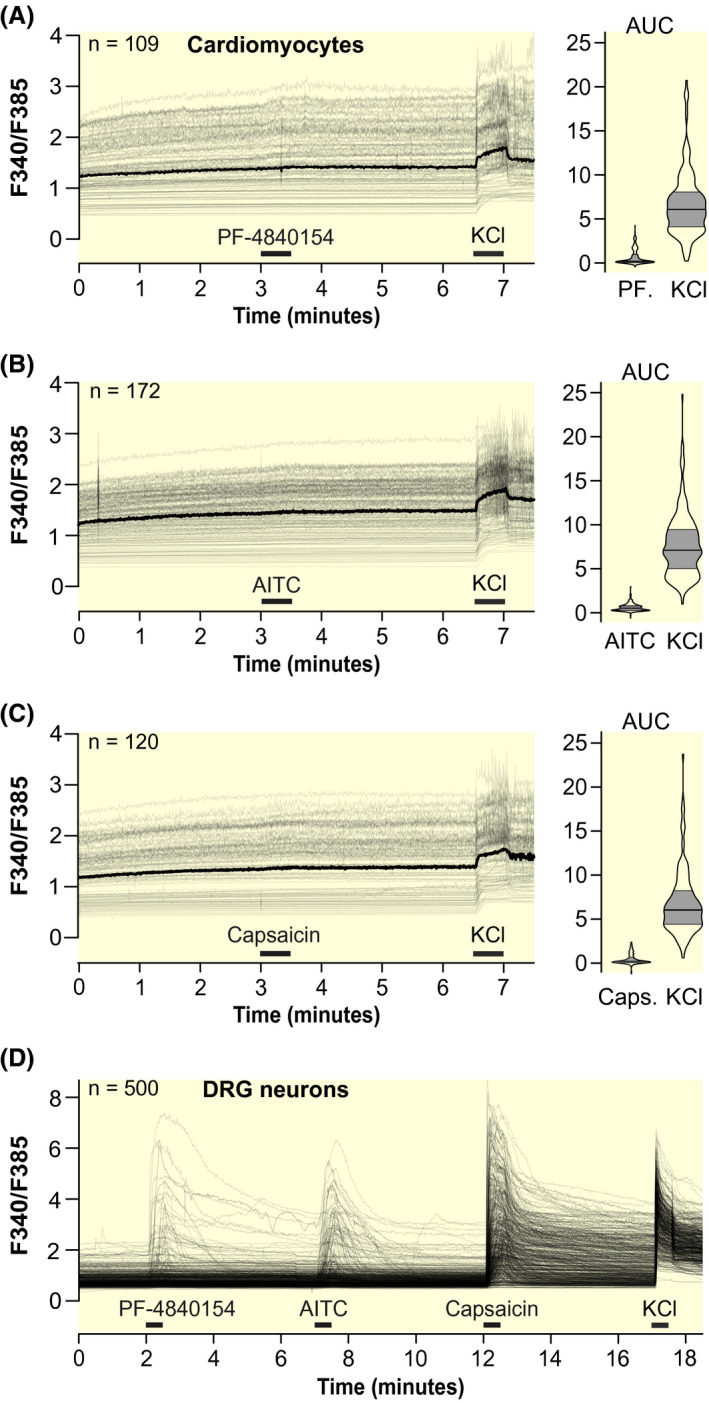
TRPA1 or TRPV1 activation elicits no relevant calcium transients in primary murine cardiomyocytes. A‐C, Individual intracellular calcium level traces of freshly isolated primary mouse cardiomyocytes, measured by fura‐2 microfluorimetry. Traces are visualized in high transparency, therefore the overlay of multiple traces results in darker grey values; the median is visualized in black. Horizontal bars indicate the application periods of TRPA1 agonists PF‐4840154 2 µmol L^‐1^ and AITC 100 µmol L^‐1^, TRPV1 agonist capsaicin 1 µmol L^‐1^, and KCl 60 mmol L^‐1^. Right panels: Violin plots of the area under the curve (AUC) of the agonist and KCl application periods. A thick horizontal line indicates the median, while the grey areas represent the second and third quartile. D, Responses of mouse DRG neurons to the TRP channel agonists used in panel A‐C and to KCl. All panels contain cells of 5 dishes of at least three animals with experiments performed on independent days. The number of cells is indicated in the upper left corner of each panel

In the first functional experiment, it was tested whether primary mouse cardiomyocytes respond to the TRPA1 agonists PF‐4840154, AITC, and JT010 and to the TRPV1 agonist capsaicin. Cardiomyocytes stained with fura‐2 were constantly superfused with extracellular solution and exposed to the respective agonists for 30 seconds. Only cells that responded to a KCl stimulation, indicating viability, were selected for analysis. The full visualization of all cells shows that there were no cardiomyocytes responding to a relevant extent to PF‐4840154, AITC (Figure 1A,B) or JT010 (Figure S1). Similarly, there were also no responses to capsaicin in mouse cardiomyocytes (Figure 1C). Responses of primary mouse DRG and TG neurons to PF‐4840154, AITC, and capsaicin serve as positive control (15%, 27%, 85% of DRG neurons and 21%, 29%, 30% of TG neurons responded with calcium increases, respectively; Figure 1D and Figure S5), whereas TG neurons from TRPA1^−/−^ mice serve as negative control (1%, 2%, 18% of TG neurons from TRPA1^−/−^ animals responded with calcium increases, respectively; Figure S5A). TRPA1 and TRPV1 have a high calcium conductance; the raw traces of responding DRG neurons show the expected large response amplitudes and demonstrate that even a low functional expression of these ion channels in cardiomyocytes would be hard to miss.

### 2.2 No evidence for functional TRPA1 or TRPV1 in electrically paced murine cardiomyocytes

It was reported that TRPA1 alters cardiac action potentials. In cardiomyocytes, contraction is the result of an intracellular calcium rise well beyond the threshold of calcium‐induced TRPA1 activation of 225‐1000 nmol L^‐1^.^29^, ^30^, ^31^ To exclude a role of TRPA1 only detectable during these short high calcium levels, electrical pacing of cardiomyocytes was established. A reliable repetitive stimulation with minimal current in the same microscope was optimized (Figure S2); area under the curve of calcium transients is analysed (Figure S3).

**FIGURE 2:**
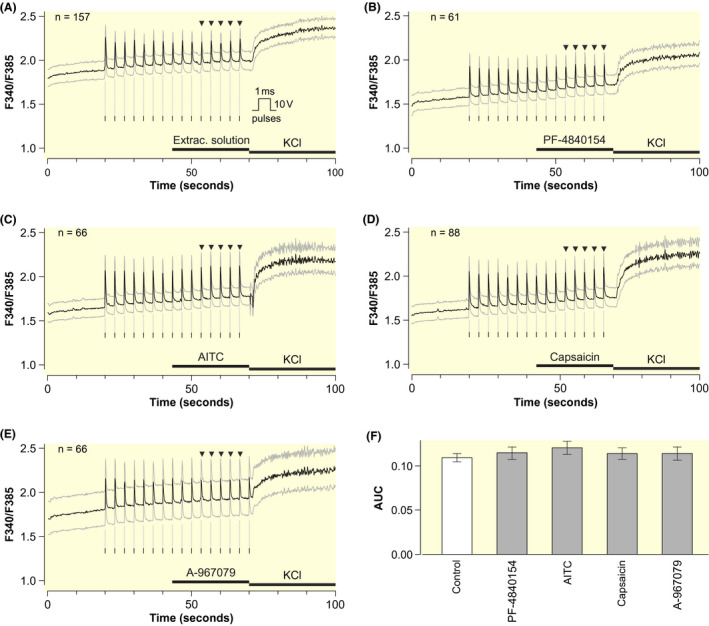
Electrically induced calcium transients in primary mouse cardiomyocytes are not affected by TRPA1 and TRPV1 modulation. A‐E, Intracellular calcium in freshly isolated cardiomyocytes. Electrical stimulation with rectangular pulses of 10 V and 1‐ms pulse duration (visualized by the inset) were delivered at a rate of 0.3 Hz as indicated by vertical lines. Traces show mean (black) ± SEM (grey). Horizontal bars indicate the application periods of control extracellular solution (same solution, but with switch to a separate perfusion channel), PF‐4840154 2 µmol L^‐1^, AITC 100 µmol L^‐1^, capsaicin 1 µmol L^‐1^, A‐967079 10 µmol L^‐1^, and KCl 60 mmol L^‐1^, which was used to verify cell viability. The AUCs of the five stimuli indicated by the black triangles were compared between the five conditions after statistical adjustment for the following nuisance parameters: (i) the intracellular calcium concentration at the beginning of the quadratic polynomial as described in Figure S3, (ii) the AUC‐values of stimuli 3‐7 and (iii) the AUC during the application of KCl. Cells were isolated from two mice, cell numbers derived from at least two dishes per experimental day are indicated in the upper left corner of each panel. F, Least square AUC mean estimates of the five marked stimuli with 95% confidence intervals for each condition. These can be interpreted as the means if all cells had exactly the same values regarding all nuisance parameters

As above, there was no relevant cytosolic calcium elevation in response to PF‐4840154  , AITC  , and capsaicin  , nor did these substances alter the shape of the electrically induced calcium transients (Figure 2). The AUC values of calcium transients during the application of test solutions were not different (*P* = .14). Importantly, this applies also to the TRPA1 antagonist A‐967079  , excluding a role of an endogenous TRPA1 activation.

### 2.3 No evidence for functional expression of TRPA1 or TRPV1 in cardiac cell lines HL‐1 and H9c2

**FIGURE 3:**
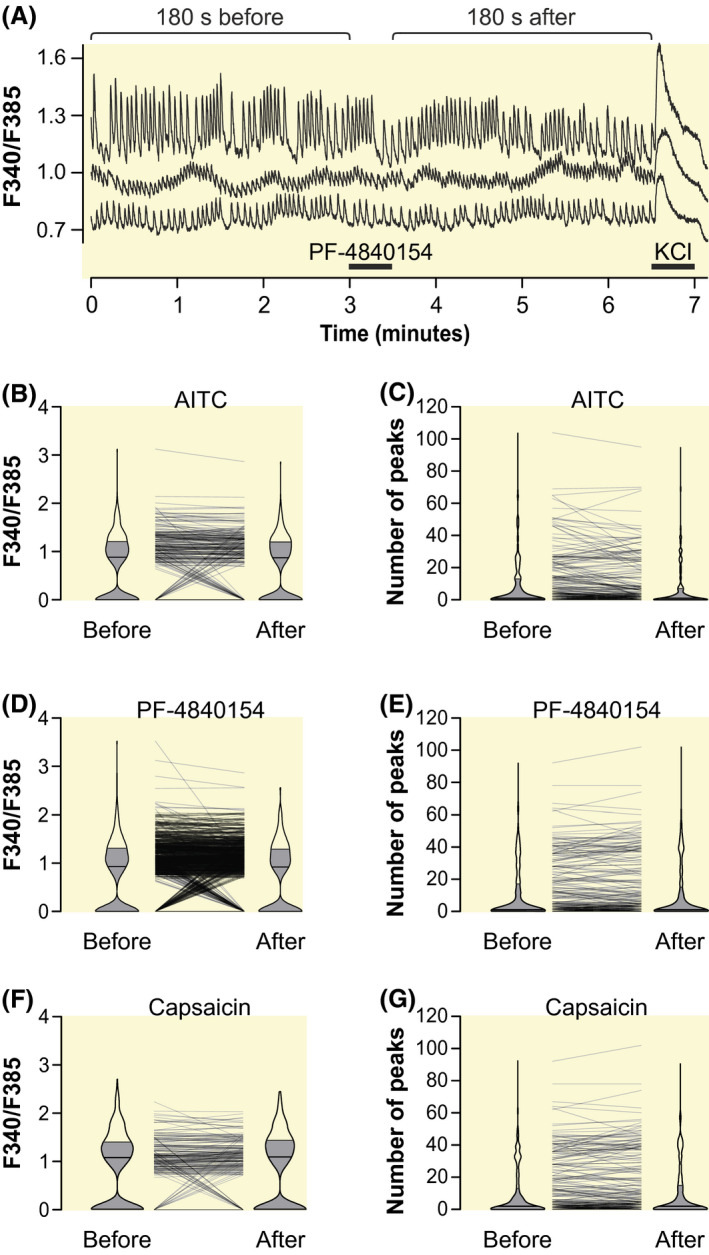
TRPA1 and TRPV1 agonists do not affect spontaneous activity of the cardiac cell line HL‐1. A, Experimental protocol with three HL‐1 cardiomyocytes exposed to PF‐4840154 2 µmol L^‐1^ as specimen. B, Amplitudes of spontaneous calcium oscillation in HL‐1 cells. An interval of 180 s before and after AITC 100 µmol L^‐1^ application was analysed. C, Number of spontaneous calcium peaks of HL‐1 cells in the 180s before and after application. D and E, Amplitudes and number of spontaneous calcium peaks before and after 30 s of PF‐4840154 2 µmol L^‐1^ application. F and G, Amplitudes and number of spontaneous calcium peaks before and after 30 s of capsaicin 1 µmol L^‐1^ application. Experiments are from 4 dishes per condition performed on 3 independent days, cell counts for stimulation with AITC, PF‐4840154 and capsaicin are 299, 279, and 277, respectively

The functional expression of TRPA1 and TRPV1 was also tested in the murine cardiac cell line HL‐1, which exhibits spontaneous contractions and intracellular calcium waves. Intracellular calcium of HL‐1 cells loaded with fura‐2 was not affected by the application of AITC, PF‐4840154, and capsaicin for 30 seconds. As before, a response to a subsequent 30 second depolarization by KCl   was used to select viable cells. Calcium transients were identified by their peaks. The calcium wave amplitudes and frequencies in the 3 minutes before the TRP agonist application were not different compared with the 3 minutes after application (Figure 3).

**FIGURE 4:**
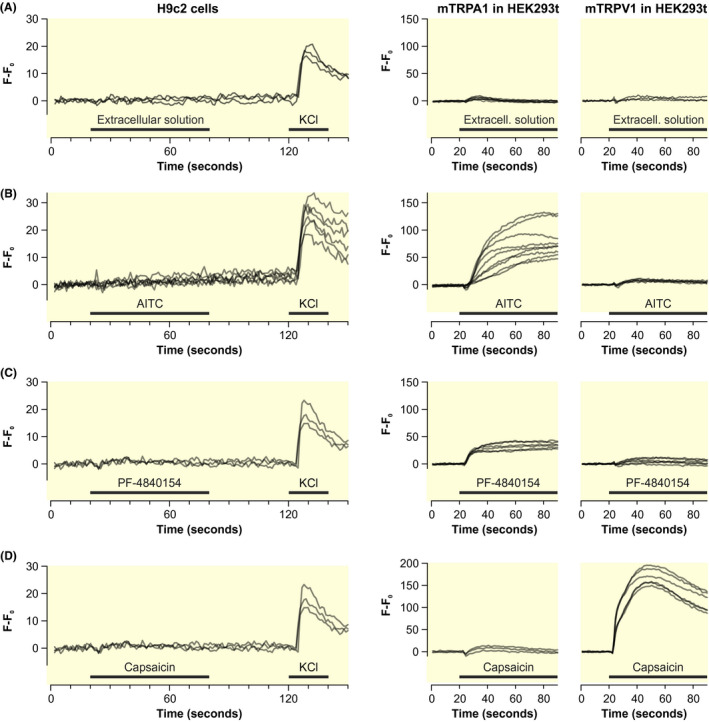
No evidence for functional TRPA1 or TRPV1 expression in the rat myoblast cell line H9c2. A, Time course of intracellular calcium levels. H9c2 cells show no response to an addition of extracellular solution (negative control), but in response to depolarization by KCl 60 mmol L^‐1^ (positive control) applied at the end of each measurement. Every trace reflects the measurement of a separate well. Right panel shows the calcium time course of HEK293t cells expressing mouse TRPA1 or mouse TRPV1, stimulated by the same substance as in the left panel. B and C, Time course of calcium traces in H9c2 and transfected HEK293t cells in response to AITC 100 µmol L^‐1^ and PF‐4840154 2 µmol L^‐1^. Right panels show responses of mouse TRPA1 or mouse TRPV1 as in panel A. D, Corresponding experiment in H9c2 and transfected HEK293t cells in response to capsaicin 1 µmol L^‐1^

The rat myoblast cell line H9c2 also shows no functional expression of TRPA1 or TRPV1. Calcium 6 fluorescence as index for intracellular calcium was measured in a fluorescent imaging plate reader with integrated pipettor. In contrast to HEK293t cells transfected with mouse TRPA1, H9c2 cells did not show a response to AITC or PF‐4840154. Similarly, mouse TRPV1‐expressing HEK293t cells respond to capsaicin but not H9c2 cells (Figure 4).

### 2.4 No evidence for relevant mRNA expression of TRPA1 or TRPV1 in murine cardiomyocytes, cardiac cell lines and human heart

**FIGURE 5:**
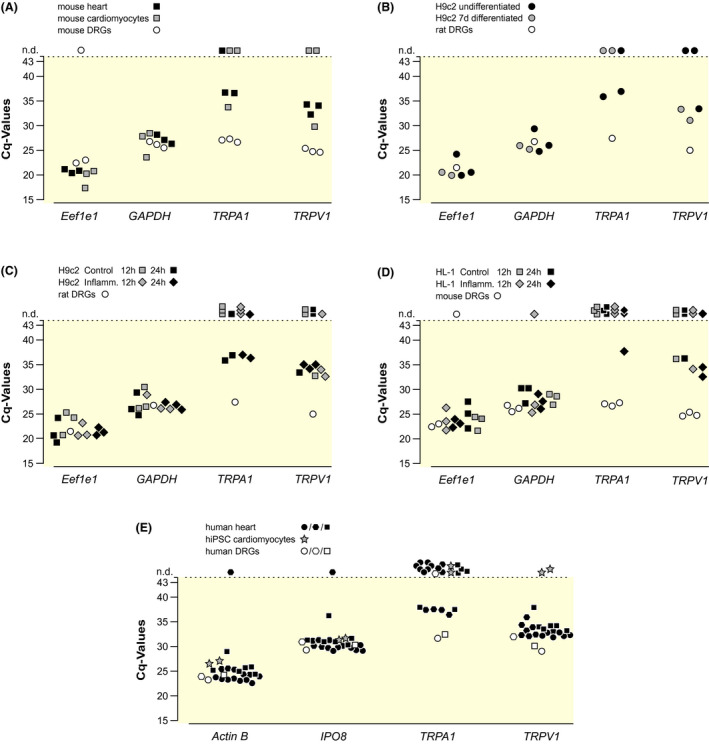
At best marginal TRPA1 or TRPV1 expression by quantitative PCR (qPCR) in cardiac tissue and cell lines. A, Isolated mouse cardiomyocytes and whole mouse hearts show higher Cq values for TRPA1 and TRPV1 than whole mouse dorsal root ganglions (DRGs). B, Cq values of H9c2 cardiomyoblasts show orders of magnitude lower TRPA1 and TRPV1 signal than rat DRGs. Differentiation by retinoic acid does not alter the expression. C, Exposure of H9c2 cells to inflammatory mediators also does not increase TRPA1 and TRPV1 expression. Control H9c2 cells for differentiation and inflammation were the same. D, Cq values of HL‐1 cardiac cell line, which similarly show orders of magnitude lower TRPA1 and TRPV1 expression than mouse DRGs, and inflammation does not alter the signal. E, Samples from seven areas of human hearts and human induced pluripotent stem cell‐derived cardiomyocytes (hiPSC cardiomyocytes) show higher Cq values for TRPA1 than human DRGs. The three human donors are separated by circles, hexagons and squares. Human heart samples and DRGs were extracted less than 10 h post‐mortem. All runs had 43 cycles, curve fitting of the cycler extrapolates to 44 cycles, as indicated by a minor tick on the y‐axis. n.d. indicates that no cDNA was detected. In all panels every symbol corresponds to a biological sample (average of two technical replicates, which had a median difference of 0.14 cycles and an interquartile range of 0.07‐0.28 cycles; Figure S4)

TRPA1 and TRPV1 mRNA levels in freshly isolated mouse cardiomyocytes and mouse heart digestions were minimal, as suggested by the high quantification cycle (Cq) values compared with DRGs, despite in the latter only a fraction of cells is positive for TRPA1 and/or TRPV1 (Figure 5A). In line with these results, Cq values for TRPA1 and TRPV1 in the H9c2 and HL‐1 cardiac cell lines were much higher than in rat and mouse DRGs. Inflammation or differentiation by retinoic acid, the latter only applied to H9c2 cardiomyoblasts, did not alter the Cq values (Figure 5B‐D). In addition, TRPA1 and TRPV1 seem to be at best marginally expressed in human cardiac tissue. In human‐induced pluripotent stem cell (hiPSC)‐derived cardiomyocytes, which have a validated cardiac phenotype in a wide range of assays,^32^, ^33^, ^34^ there was no detectable TRPA1 or TRPV1 mRNA (Figure 5E). In an additional control, TRPA1 mRNA expression was marginal in TG of TRPA1^−/−^ mice compared with wild type animals, whereas the TRPV1 expression was similar in both (Figure S5B).

Further, we aimed to investigate TRPA1 protein levels in the heart. It is well known that antibodies for the detection of TRPA1 have been particularly troublesome.^35^ Despite other antibodies, we also chose anti‐mTRPA1 (ARP35205_P050, Aviva Systems), which was validated in TRPA1 knockout mice.^36^ We first tested this antibody on HEK293t cells expressing mTRPA1‐ires‐YFP. The fluorescent signal corresponding to the anti‐mTRPA1 antibody was not positively correlated with the fluorescent reporter (data not shown). In addition, we tried to validate the antibody by functional responses by probing cultured mouse DRGs with JT010 and fixing them immediately thereafter. However, the anti‐mTRPA1 antibody staining was not positively correlated with the calcium transients evoked by the TRPA1 agonist. Furthermore, this antibody generated a fluorescent signal in dorsal root ganglion neurons from a TRPA1^−/−^ mouse. Finally, also staining of mouse DRGs did not detect a subpopulation of neurons in accordance with the literature. With these four approaches questioning the credibility of one of the few validated antibodies, we decided not to report the extensive attempts to provide structural insights using immunohistochemistry.

## 3 DISCUSSION

The main finding of this study is that there is no functional TRPA1 and TRPV1 in murine cardiomyocytes. The finding is based on converging evidence from a complete lack of functional responses to stimulation with specific agonists of primary mouse cardiomyocytes and cardiomyocyte cell lines and the absence of relevant mRNA amounts.

Why was this study performed? The original aim was to understand the role of TRPA1 for cardiac physiology and pathophysiology, irrespective of its location on cardiomyocytes, the supplying sensory neurons, or other resident cell types. This aim included identifying potential cardiac side effects of TRPA1 agonists and antagonists that might be used as future pain medication, as well as therapeutic strategies for cardiovascular diseases. Protocols to detect the respective site of action were designed, including tissue‐specific knockouts, assuming that there is, based on the literature, a TRPA1 response of cardiomyocytes. However, when cardiomyocytes were tested with established and the most potent TRPA1 agonists, there was the unexpected result of the absence of responses. This was then corroborated with several approaches, including the repetition of reported experiments.

Unravelling the role of TRPA1 in cardiac physiology and pathophysiology is easier in case TRPA1 is found in fewer cell types, and this applies in particular to the absence of functional TRPA1 in cardiomyocytes. However, the discrepancy of our findings with prior publications of more than one group requires a consideration in detail.

### 3.1 Functional findings indicating the absence of TRPA1 and TRPV1 in cardiomyocytes

Our results rule out a biologically relevant amount of functional TRPA1 ion channels in cardiomyocytes isolated from wild type C57BL/6 mice, which is in discrepancy to other published data.^21^ However, our findings are based on a larger number of cardiomyocytes and cardiac cell lines. These cells showed large responses to potassium‐induced depolarization used as a positive control, but did neither show any relevant calcium transients in response to the two selective and potent TRPA1 agonists PF‐4840154 and JT010, nor to the less specific but established TRPA1 agonist AITC. In addition, the lack of responses to capsaicin suggests the absence of functional TRPV1 ion channels. Technical reasons for a failure were excluded by the responses of DRG and TG neurons and of cells expressing TRPA1 or TRPV1 to the mentioned agonists and lack of responses to TRPA1 agonists of TRPA1^−/−^ neurons. Thus, the report by Andrei et al.^21^ showing an intracellular calcium increase in primary mouse cardiomyocytes challenged with TRPA1 activators remains unclear. Their number of measured cells (eg., in their figure 5) is not given, but even a low cell count would not constitute a plausible explanation for these fundamentally different findings. Also, the following paper of the same group^20^ contains calcium measurements upon pharmacological stimulation. As agonist, only AITC has been used, and only a single specimen of one cell responding to AITC is shown. Unfortunately, this representative cell showed no particularly stable behaviour before AITC application. This applies also to the TRPA1 knockout, where only one cardiomyocyte as a specimen is depicted.

In addition, the mentioned paper shows five subsequent calcium transients in response to electrical stimulation but only from one cardiomyocyte and cut out of context.^20^ The measurements are limited to a total of 20 cells in this approach, while we provide more than threefold for two agonists. Despite mentioning a superfusion device, they indicated applications in the form of arrows and not horizontal bars, and increasing concentrations were not measured in separate cells, but AITC was added to the bath in a cumulative fashion to the same cells without washout periods. In particular, the long duration of the experiment inevitably resulting from the cumulative concentrations used in Andrei et al.^20^ might have resulted in critical cumulation of reactive chemicals associated with the electrostimulation, as demonstrated in the present Figure S3. This might have appeared as an agonist‐induced change but cannot be assessed by the reader because only single specimens were presented without context.

To address their claim that ‘TRPA1 ion channel stimulation enhances cardiomyocyte contractile function’, electrically paced cardiomyocytes were exposed to TRPA1 agonists to exclude a relevance of the channel only at the high calcium levels associated with action potentials. Extra care was taken to use stimulation parameters at which the electrical stimulation does not generate a calcium‐elevating amount of reactive chemical species. In our hands, a concentration of 100 µmol L^‐1^ AITC, shown to increase calcium transients in Andrei et al.,^20^ did not cause any calcium transient change of electrically paced cardiomyocytes. Moreover, a more potent and specific TRPA1 agonist, PF‐4840154, also failed to elicit any calcium transient changes in electrically paced cardiomyocytes. Analogous to previous experiments, the cells exhibited intracellular calcium level increases to KCl, demonstrating that they had the ability to respond. Besides showing the absence of effects of different TRPA1 agonists, our results are further strengthened by rigorous experimental design, including a higher total cell count than previously presented. Additionally, experiments were repeated on separate animals and days.

The HL‐1 and H9c2 cell lines are a common model to study cardiomyocytes. HL‐1 cells spontaneously depolarize; therefore, one would expect alterations of frequency or amplitude of calcium transients in response to TRPA1 activation. However, there were no such changes after AITC, PF‐4840154, or capsaicin application. The cardiomyoblast cell line H9c2 has no spontaneous calcium oscillations, which rendered it suitable for mass cell imaging in the fluorescent imaging plate reader with integrated pipettor. Although the TRPA1 agonists elicited pronounced responses in TRPA1‐transfected HEK293t cells, they failed to do so in H9c2 cells. Similarly, capsaicin activated TRPV1 expressed in HEK293t cells, but elicited no calcium transients in H9c2 cells. Taken together, both common cardiac cell lines HL‐1 and H9c2 seem not to express TRPA1 or TRPV1, and this can be considered supportive for the results in primary cardiomyocytes.

There are additional publications claiming functional TRPA1 expression in cardiomyocytes. Conklin et al.^19^ provided functional data using patch clamp electrophysiology, with increased open probability stimulated by TRPA1 agonist cinnamaldehyde and inhibition by HC‐030031. This is a challenging and respected technique, but the dataset is rather limited. A total of nine traces from an unknown number of cells ‘representative of two independent experiments’ are shown. The different noise levels suggest that the recordings are from different cells. Therefore, intercell variability adds to the observation, and an increased open probability due to agonist application, which is reduced again by the antagonist in the same cell, has not been demonstrated. Further, the reported open probability change from nPo = 0.03 in control to 0.09 with cinnamaldehyde is not large, considering that the latter is a full agonist. In contrast, their calcium imaging with TRPA1 agonist acrolein at concentrations of 10 and 25 µmol L^‐1^ failed to detect a calcium transient in response to the application. Why a calcium change 25 minutes after acrolein application was considered acrolein‐dependent is unclear and cannot be accepted as the activation of a calcium‐permeable ion channel.

Andrei et al.^21^ also reported responses of cardiomyocytes to TRPV1 agonist capsaicin 100 nmol L^‐1^, which were absent in the respective knockout and inhibited by TRPV1 antagonist SB366791. This conflicts with our lack of functional responses to capsaicin in primary cardiomyocytes as well as in cardiac cell lines, also backed up by positive controls using sensory neurons and cells expressing mouse TRPV1. More importantly, a TRPV1‐Cre Td‐tomato reporter mouse model also found no evidence for TRPV1 in cardiomyocytes.^28^

### 3.2 Investigations of TRPA1 mRNA and protein in cardiac cells

The results indicate that there is, at best, a minimal amount of TRPA1 mRNA in cardiac tissue, primary cardiomyocytes, or cardiac cell lines. This is in contrast to previous reports, which describe the expression of these channels in primary cardiomyocytes, cardiac tissue, and the H9c2 cardiac cell line.^17^, ^18^, ^19^ However, in Conklin et al.,^19^ the Cq values are only given as delta‐Cq to the housekeeping gene *ActinB*, whose Cq value is not mentioned and which is the only housekeeping gene tested. Nevertheless, their mean delta‐Cq value is 15.8 in cardiomyocytes and 4.6 for DRGs. Assuming a qPCR efficacy of about 2, this indicates a TRPA1 expression 2352‐fold lower in cardiomyocytes compared with DRGs. Their TRPA1 levels in DRGs relative to the house‐keeping gene seems in line with our results. Also, in Pazienza et al.,^18^ the TRPA1 expression is only mentioned in arbitrary units. No results were reported for their housekeeping genes, which precludes a direct comparison with our results. They report a higher signal in cardiomyocytes compared with non‐cardiomyocytes. A hypothesis that might explain mRNA detected in heart samples would be nerve terminals containing TRP channel mRNA, which might remain attached to the cardiomyocytes in isolation procedures. In Lu et al.,^17^ the Cq values of cardiomyocytes, left ventricular cardiac tissue, and H9c2 cells are around 30, which places them about 8‐10 cycles above GAPDH, which is not far from our observations. Overall, the mentioned papers have put emphasis on finding some TRPA1 mRNA and fall short on a critical discussion of how small this signal was. We would rather conclude that, for example, the mentioned TRPA1 expression below 1:1000th in cardiomyocytes compared with DRGs could be considered marginal, which fits the functional data that show no responses.

Furthermore, there is at best a minimal amount of the TRPV1 channel expressed in primary mouse cardiomyocytes, mouse cardiac tissue, and cardiac cell lines, even though TRPV1 Cq values are below those of TRPA1. It is unclear why in the human samples TRPV1 Cq values of cardiac tissue are closer to DRGs and whether a different rate of post‐mortem RNA degradation could contribute to this. The post‐mortem RNA degradation overall increases Cq values, which leads to more nondetected values and makes comparison of tissues harder. In summary, the TRPV1 expression in DRG neurons was higher than in cardiomyocytes.

It has been described that TRPA1 and TRPV1 expression in sensory neurons and other cell types are upregulated by inflammatory mediators.^37^, ^38^, ^39^, ^40^ However, no such change in mRNA expression was observed upon exposure of HL‐1 and H9c2 cells to such an ‘inflammatory soup’.^41^ As an additional attempt to upregulate mRNA expression of TRPA1 and TRPV1, we differentiated the cardiomyoblast cell line H9c2 by the application of retinoic acid as previously described.^42^, ^43^ This treatment did not affect TRPA1 or TRPV1 mRNA levels, which suggests that the expression of these channels is not induced by differentiation. To ensure the quality of our qPCR experiments, we selected two reliable housekeeping genes, which showed similar Cq values across all samples of one species. As control for the functional measurements in TG neurons, the expected contrast in TRPA1 mRNA between wild type and TRPA1^−/−^ neurons was verified, and no contrast in TRPV1 mRNA or the house keeping genes was observed. Even if this is widely known, a note of care has to be attached to mRNA data. Messenger RNA can be alternatively spliced and the translation can be inhibited. Its added value in the current context comes from the rather limited qualities of anti‐TRPA1 antibodies.

It is well known that antibody‐based detection is particularly problematic for TRPA1, in blots and in immunohistochemistry. A study was dedicated to the quality of five commonly used anti‐human TRPA1 antibodies, which have been carefully tested in western blots and immunocytochemistry.^35^ That study validated TRPA1 antibodies 6G8 and C‐5, which were not used by the groups reporting TRPA1 in cardiomyocytes. In contrast, the study invalidated NB110‐40763, which labelled also other proteins, and in particular Ab58844 and ACC‐037, both labelling only antigens other than human TRPA1. This calls the use of these antibodies to detect TRPA1 into question. In Andrei et al.,^21^ it is reported that TRPA1 is localized in costameres and Z‐discs of cardiomyocytes, using immunohistochemistry and immunocytochemistry. Nonetheless, the information provided for the polyclonal antibody does not uniquely identify one of the six antibodies manufactured by Novus Biologicals. Also in Lu et al., one of the Novus Biologicals TRPA1 antibodies is used without providing further details.^17^ Only the supplier's website provides their paper as reference for the NB110‐40763 antibody. A TRPA1^‐/‐^ control, a secondary antibody control or a blocking peptides control, was not reported. In Conklin et al.,^19^ the antibody ACC‐037 was used to detect TRPA1 in murine left ventricles and murine cardiomyocyte membrane by blotting, as well as in murine isolated cardiomyocytes, paraffin sections of noninfarcted human cardiac tissue, and a mouse heart by immunohistochemistry, and the antibody NB110‐40763 was used to stain isolated cardiomyocytes.^19^ Despite some controls, these antibodies are rather promiscuous and unspecific, which questions their findings regarding TRPA1 expression at a protein level and its localization in cardiac tissue.^35^ A TRPA1 reporter model is in development, which has so far only been published as a conference abstract, and will hopefully contribute to solving this issue. Although initially planned, we finally abstained from including antibody‐based evidence, as we did not find a viable antibody for rodent TRPA1. The TRPA1 knockout‐validated ARP35205_P050 antibody from Aviva Systems Biology (used in Nassini et al.^36^) was tested, but the staining of this antibody was not correlated with the fluorescent signal coming from a mTRPA1‐ires‐YFP plasmid, nor correlated to response to TRPA1 agonists. Moreover, the antibody generated a fluorescent signal in dorsal root ganglion neurons from a TRPA1^‐/‐^ specimen.

Potential limitations of our study should be mentioned. Our results do not necessarily apply to other than the investigated species. Human TRPA1 mRNA degrades after death, and it cannot be fully excluded that this leads to a false negative result in the post‐mortem samples. The three human donor samples might not be representative of the population, for example, due to age. Further, the laboratory animals used in this study are an inbred strain. It is theoretically possible that other strains have cardiomyocytes expressing TRPA1. In cell lines, differentiation and inflammation were tested, which does not fully exclude a TRPA1 expression in pathophysiological states as, for example, diabetes or hypertrophy. Further, although considered unlikely, it is principally conceivable that cell culture leads to a loss of functional TRPA1.

Nevertheless, our data strongly support the view that TRPA1 is not functionally expressed in mouse cardiomyocytes and probably also not in human cardiomyocytes. Also in *Drosophila melanogaster,* TRPA1 had to be overexpressed in the heart to investigate effects.^44^ Conclusions from evolutionary distant species might be limited, as mammalian TRPA1 is activated by cool temperatures and inactivated by heat, and the reverse is true for fly TRPA1.^45^

It was not our primary aim to falsify the work of others, but the overall dataset from independent laboratories had motivated us to build on their work. However, in previous publications reporting the opposite, the amount of presented functional data is limited, and none of the newer and more potent agonists have been used. Previous claims were supported by an incomplete reporting of TRPA1 mRNA quantification and binding of antibodies erroneously assumed to be specific for TRPA1. TRPA1 remains an interesting drug target, and for this, a direct effect on cardiomyocytes would have been of concern. In light of our results, this concern appears unfounded.

## 4 MATERIALS AND METHODS

### 4.1 Chemicals and solutions

The extracellular solution used for cellular experiments contains (in millimolar) 145 NaCl, 5 KCl, 10 glucose, 10 4‐(2‐hydroxyethyl)piperazine‐1‐ethanesulfonic acid (HEPES), 1.25 CaCl_2_, and 1 MgCl_2_ , buffered to pH 7.4 with NaOH and has an osmolarity of 300 mosm. The Ca^2+^ free solution used to isolate cardiomyocytes contains (in millimolar) 134 NaCl, 11 glucose, 4 KCl, 1.2 MgSO_4_, 1.2 Na_2_HPO_4_, 10 HEPES. This solution was buffered to pH 7.35 with NaOH, and the myosin inhibitor 2,3‐butanedione monoxime 10 mmol L^‐1^ was added. Abovementioned salts and HEPES were obtained from Carl Roth (Karlsruhe, Germany), Sigma‐Aldrich (St. Louis, MO, USA), or Merck (Darmstadt, Germany) and NaOH from Thermo Fisher scientific (Waltham, MA, USA). PF‐4840154 was obtained from MedChemExpress (Monmouth Junction, NJ, USA), JT010, AITC, A‐967079, capsaicin, all‐trans retinoic acid and 4′,6‐diamidino‐2‐phenylindole (DAPI) were obtained from Sigma‐Aldrich. The inflammatory soup was composed of bradykinin (Santa Cruz Biotechnology, Dallas, TX, USA), serotonin (Fluorochem, Hadfield, UK), histamine (Acros organics, Fair Lawn, NJ, USA) and prostaglandin E2 (AdipoGen, San Diego, CA, USA) at a concentration of 10 µmol L^‐1^ each. Culture additives l‐carnitine, creatine, taurine, nonessential amino acids, insulin–transferrin–selenium, and linoleic‐oleic acid were obtained from Gibco and Sigma‐Aldrich.

### 4.2 Isolation of primary cardiomyocytes and dorsal root ganglion neurons

Breeding, euthanasia, and all procedures of animal handling were performed according to regulations of animal care and welfare. Experiments were carried out in accordance with the European Communities Council Directive of 24 November 1986 (86/609/EEC) and approved by the respective ministry (BMBWF‐66.009/0413). Wild type C57BL/6, TRPA1^−/−^ mice (generated by Kwan et al.^7^) and Sprague‐Dawley rats (13‐17 weeks of age) were killed by cervical dislocation, preceded by anaesthesia by an exposure to isoflurane or rising CO_2_ levels.

For isolated primary cardiomyocytes, a volume of 5 mL of ice‐cold calcium‐free solution was injected into the ventricles to clean them from blood and stop the contractions, as previously described.^46^ Hearts were surgically removed, suspended in the Langendorff apparatus by the aorta, retrogradely perfused at 37°C with a calcium‐free solution for 3 minutes and then with a calcium‐free solution containing 0.17 mg mL^−1^ Liberase (Roche, Manheim, Germany) for 18 minutes at 180 mL min^−1^. The atria were discarded, and the ventricles were mechanically triturated with fine forceps. The cellular digestion solution was then incubated on a shaker at 37°C and was progressively exposed to increasing concentrations of calcium, reaching a maximum of 0.2 mmol L^‐1^ of calcium over 1 hour in five steps. A final step of mechanical trituration with a plastic Pasteur pipette was performed; isolated cardiomyocytes were centrifuged at ​ 55 *g* for 2 minutes and resuspended with a glass pipette in Minimal Essential Medium Eagle alpha (M8042 with 1.8‐mmol L^‐1^ calcium, Sigma‐Aldrich) supplemented with 10 µg mL^−1^ insulin, 5.5 µg mL^−1^ transferrin, and 5 ng mL^−1^ selenium, penicillin/streptomycin 1%, l‐glutamine 1% (all Sigma‐Aldrich), and (−)‐blebbistatin 180 µmol L^‐1^ (Cayman Chemical, Ann Arbor, MI, USA). Isolated cardiomyocytes were used directly for RNA isolation or plated on either 35 mm dishes or 12 mm coverslips coated with Matrigel Membrane Matrix 11543550 diluted at 1/10 (Corning, Corning, NY, USA) and cultured at 37°C and 5% CO_2_. All experiments were performed 1 hour after isolation to ensure cardiomyocyte recovery and attachment to Matrigel.

For cultures of sensory neurons, DRGs from all spinal levels or TGs were excised and either used directly for RNA isolation or transferred to DMEM (D5648, Sigma‐Aldrich) containing streptomycin/penicillin 1% (Lonza, Basel, Switzerland) and l‐glutamine 1% (Lonza), treated with 1 mg mL^−1^ collagenase (Sigma‐Aldrich) and 3 mg mL^−1^ Dispase II (Roche) for 60 minutes at 37°C. Digested DRGs or TGs were then mechanically dissociated with a Pasteur pipette, centrifuged at 232 *g* for 5 minutes, and plated onto 12 mm glass coverslips previously coated with poly‐d‐lysine (100 mg mL^−1^, Sigma‐Aldrich). DRG and TG neurons were cultured in DMEM supplemented with streptomycin/penicillin 1% (Lonza), l‐glutamine 1% (Lonza), and mouse nerve growth factor 100 ng mL^−1^ (Alomone Labs, Tel Aviv, Israel). Neurons were cultured at 37°C and 5% CO_2_ for 15‐30 hours.

### 4.3 HL‐1 and H9c2 cell lines

The HL‐1 cell line was received from Dr Dirk von Lewinski (Division of Cardiology, Medical University of Graz, Austria) and was maintained following the protocol recommended by Sigma‐Aldrich. HL‐1 cells from passages 52‐60 were seeded in gelatin/fibronectin (both Sigma‐Aldrich) coated flasks and cultured in Claycomb basal medium (Sigma‐Aldrich) supplemented with HL‐1 Qualified FBS F2442 10% (Sigma‐Aldrich), norepinephrine 0.1 mmol L^‐1^ (Sigma‐Aldrich), l‐ascorbic acid 30 mmol L^‐1^ (Sigma‐Aldrich), l‐glutamine 2 mmol L^‐1^ (Lonza), and penicillin/streptomycin 1% (Lonza). The medium was replaced every day and HL‐1 cells were split in a 1:3 ratio every three days. For splitting, cells were washed in Hank's balanced salt solution (HBSS) without calcium and magnesium, detached using trypsin‐EDTA (T3924, Sigma‐Aldrich) and centrifuged at 300 g for 2 minutes in HBSS. For single‐cell imaging, 37 500 to 75 000 HL‐1 cells were seeded on gelatin/fibronectin‐coated 12 mm glass coverslips.

The myoblast cell line H9c2 (H9c2(2‐1)) was obtained from ATCC (Manassas, VA, USA). Cells were seeded in cell culture flasks in M199 medium (M5017, Sigma‐Aldrich) supplemented with l‐glutamine 1% (Lonza), penicillin/streptomycin 1% (Lonza), and fetal bovine serum 10% (Sigma‐Aldrich). H9c2 cells were subcultured at 60%‐70% confluence and split in a 1:2 ratio. For splitting, cells were washed using HBSS without calcium and magnesium, detached using trypsin‐EDTA (Lonza), and centrifuged at 232 *g* for 5 minutes in HBSS. All cell lines were cultured at 37°C with 5% CO_2_. For Fluorescent Imaging Plate Reader calcium assays, 30 000 H9c2 cells were seeded per well in a black F‐bottom 96‐well cell culture plate (Greiner Bio‐One, Kremsmünster, Austria) coated with poly‐d‐lysine (100 mg mL^−1^, Sigma‐Aldrich). For differentiation of H9c2 cells, 24 hours after seeding the medium was changed to a low‐serum medium (1% fetal bovine serum), and 1 µmol L^‐1^ all‐trans retinoic acid was added daily for 7 days. Undifferentiated and retinoic acid‐differentiated cells were visually different, and in the latter, the number of multinucleated cells was substantially higher (*X* ^2^ (1, N = 1479) = 8.2, *P* = .004). For inflammatory stimulation of HL‐1 and H9c2 cell lines, cells were exposed 24 hours after seeding to either culture medium or culture medium supplied with an inflammatory soup for 12 or 24 hours. After this, RNA was immediately isolated.

### 4.4 Maturing hiPSC‐derived cardiomyocytes

Derived from human‐induced pluripotent stem cells (hiPSCs), iCell^®^ Cardiomyocytes from FUJIFILM Cellular Dynamics, Inc (Madison, WI, USA) were used. The hiPSC‐derived cardiomyocytes were thawed and plated to a density of 20 000 cells/well on 0.1% gelatin coated 96‐well plate, using iCell Cardiomyocyte Plating Medium (FUJIFILM Cellular Dynamics). The typical neonatal phenotype of human cardiomyocytes, characterized by a high level of flexibility and the ability of changing phenotypes, must be suppressed to enable meaningful experimental conditions. Therefore, maturation medium DMEM without glucose, supplemented with 2 mmol L^‐1^ l‐carnitine, 5 mmol L^‐1^ creatine, 5 mmol L^‐1^ taurine, 1 mmol L^‐1^ nonessential amino acids, 1.0 mg mL^−1^ recombinant human insulin, 0.55 mg mL^−1^ human transferrin, 0.5 μg mL^−1^ sodium selenite, 1× linoleic‐oleic acid, and 10 mmol L^‐1^ HEPES was used, as previously described, in order to generate an adult ventricular cardiomyocyte phenotype.^34^ Cells were washed and cultivated with maturation medium for 48 hours at 37°C with 7% CO_2_. The medium was then removed and replaced with iCell Cardiomyocytes Maintenance Medium (FUJIFILM Cellular Dynamics).

### 4.5 HEK293t cells and transient transfection

HEK293t cells were cultured in DMEM supplemented with fetal bovine serum 10% (Sigma‐Aldrich), l‐glutamine 1% (Lonza) and penicillin/streptomycin 1% (Lonza), at 37°C and 5% CO_2_. HEK293t cells were transiently transfected with JetPEI reagent (PolyPlus, Illkirch, France), and measurements were performed 18‐20 hours after seeding in flat bottom 96‐well plates for calcium imaging in a fluorescent imaging plate reader, or in 12 mm coverslips for immunocytochemistry. For calcium measurements, pcDNA3.1 plasmids encoding for mouse TRPA1 and TRPV1 were used, as described previously.^47^, ^48^ As a reference for immunocytochemistry, mTRPA1‐ires‐YFP was expressed in HEK293t cells.^49^

### 4.6 Single‐cell fluorescent imaging

TRPA1 has a high calcium permeability^50^; therefore, observation of cytosolic calcium by microfluorimetry appeared as a method of choice. The coverslips were incubated with fura‐2 AM ester (Biotium, Hayward, CA, USA) for 30 minutes at 37°C and 5% CO_2_, before placement in glass‐bottom 35 mm dishes in extracellular solution and a recovery period of 10 minutes. Then they were mounted onto an Olympus IX73‐inverted microscope (Olympus, Tokyo, Japan) and imaged using a 10× objective. Cells were permanently superfused with extracellular solution using a software‐controlled 8‐channel, gravity‐driven, common‐outlet system (ALA Scientific Instruments Inc, Farmingdale, NY, USA). This superfusion was switched to different solutions after adequate baseline recording. A positive control detecting viable cells was added at the end of each recording, using depolarization by an extracellular solution with 60 mmol L^‐1^ KCl (isotonic replacement of NaCl) for cardiomyocytes and sensory neurons. Fura‐2 was alternatingly excited for 30 ms by a 340‐nm LED (50 mW, used at 100%) and by a 385‐nm LED (1435 mW, used at 5%) using an Omicron LEDHub (Laserage‐Laserprodukte GmbH, Rodgau‐Dudenhofen, Germany). Fluorescence emission was long‐pass filtered at 495 nm, and pairs of images were acquired at a rate of 1 Hz with a 4.2‐MP 16‐bit CCD camera (6.5‐μm pixel edge length, 18.8‐mm sensor diameter, Prime BSI; Teledyne Photometrics, Tucson, AZ, USA). The hardware was controlled by the μManager 1.4 plugin in ImageJ.^51^ The background intensity was subtracted before calculating the ratio between the fluorescence emitted when the dye was excited at 340 and 385 nm (F340/F385 nm). The time course of this ratio was analysed for regions of interest adapted to individual cells.

For electrical stimulation, cardiomyocytes were seeded in 12 mm coverslips. Electrical pacing was optimized for reliable stimulation at minimal current flow. This was done to base stimulation on capacitive currents and to limit electrolysis with subsequent generation of reactive chemical species due to faradaic currents.^52^ This included a minimized distance of 4 mm between the platinum electrodes adapted to the optical field, allowing a high dV/ds with a low stimulation voltage. Stimulation duration at 10 V and voltage at 1 ms duration were independently varied (Figure S2). With the chosen parameters of 10 V for 1 ms at 0.3 Hz, no cellular damage was observed within the recording duration. The distance to damaging electrical stimulation parameters were explored, by incrementing the voltage in steps of 5 V. Electrical stimuli were delivered by a Biopac MP30 isolated stimulator and the respective Biopac 3.7 software.

### 4.7 Fluorescent imaging plate reader calcium assays

For fluorescent imaging plate reader calcium assays, H9c2 and transfected HEK293t cells were seeded at a density of 30 000 cells/well in a black F‐bottom 96‐well cell culture plate (Greiner Bio‐One) coated with poly‐d‐lysine (100 mg mL^−1^, Sigma‐Aldrich). Then they were loaded with the Calcium 6 dye for 2 hours (Calcium 6 Kit by Molecular devices, San Jose, CA, USA) in extracellular solution. According to the manufacturer's protocol, cells were not washed, but extracellular dye was chemically quenched. Calcium 6 fluorescence excited at 488 nm every 2.5 seconds served as index of intracellular calcium. Assays were carried out at 25°C with a fluorescent imaging plate reader with integrated pipettor (FlexStation 3, Molecular devices). A volume of 50 µL containing test substances was added automatically according to a preset protocol into 100 µL of extracellular solution in the wells, 20 seconds after the start of the measurement for agonists and 120 seconds for the positive control KCl 60 mmol L^‐1^. Fluorescence change relative to baseline fluorescence is reported (*F* − *F*_0_).

### 4.8 RNA extraction, reverse transcription and real‐time qPCR

Human hearts and DRGs were obtained according to local ethic regulations, from the cadavers of three human body donors, who had given written consent to donate their dead bodies to the Division of Anatomy of the Medical University of Vienna for teaching and science. The donors were aged 92 years (female) and 88 and 80 years (male), and the tissue was extracted less than 10 hours post‐mortem. For RNA isolation, a small piece of tissue was cut from seven regions of the human heart and one human DRG and subjected to lysis.

Mouse hearts were extracted from C57BL/6 wildtype mice in cages enriched with a running wheel. To isolate RNA, mouse DRGs and pieces of human DRGs and hearts were lysed with a Precellys 24 device (VWR, Radnor, PA, USA) in TriFast reagent (VWR) or TRIzol (Thermo Fisher Scientific), whereas RNA from the cell lines was extracted directly using the TriFast reagent or Trizol, according to the manufacturer's protocol. In short, cells were washed with PBS without Ca^2+^ or Mg^2+^, incubated in TriFast until lysed and then 200 µL chloroform (Sigma‐Aldrich) were added per millilitre. Solutions were shaken and centrifuged for 15 minutes when phases started to separate. To the supernatants, a 1.1‐fold volume of isopropanol (Merck) was added, the solution shaken, incubated for 10 minutes on ice, and centrifuged for 30 minutes. Supernatants were removed, 1 mL of 70% ethanol (Merck) was added, and reaction tubes were centrifuged for 6 minutes. After that, ethanol was removed and pellets were resuspended in 10 µL diethyl pyrocarbonate‐treated water (Thermo Fisher Scientific) at 53°C for 10 minutes in a shaker (Mixing Block MB‐102, Bioer, Hangzhou, China). All centrifugation steps were performed at 12 000 g at 4°C. RNA concentration was measured using a NanoDrop 2000c device (Thermo Fisher Scientific). Total RNA was transcribed into complementary DNA (cDNA) with the High‐Capacity cDNA Reverse Transcription Kit (Thermo Fisher Scientific) following the kit instructions. Briefly, 2 µL of 10× buffer, 0.8 µl of 25× dNTPs, 2 µL of 10× primers, 1 µL of polymerase, 4.2 µL of diethyl pyrocarbonate‐treated water (Thermo Fisher Scientific), and 10 µL of RNA (maximum concentration: 0.2 µg µL^−1^ RNA) were mixed. The reverse transcription was performed as follows: 25°C for 10 minutes, 37°C for 2 hours, and 85°C for 5 minutes, and final cooling at 4°C. The concentration of cDNA was determined by measurement with a NanoDrop 2000c device. The qPCRs were performed using the Sso Advanced Mastermix (172‐5271, Bio‐Rad, Hercules, CA, USA), according to the manufacturer's instructions. Each qPCR reaction contained 500 nM primer and 100 ng cDNA. The cycler program was set as follows: 95°C for 30 seconds followed by 43 cycles of 95°C for 15 seconds, 60°C for 30 seconds, and a final ramp from 60°C to 95°C incrementing 5°C every 5 seconds. According to the MIQE guidelines, not Ct but the term quantification cycle (Cq) is used.^53^ All Cq values calculated by the cycler software are below 44 or were visualized as ‘not detected’. Differences between the two technical replicates of all samples are given in Figure S4. Primers are summarized in Table S1.

### 4.9 Immunocytochemistry

For stainings of DRGs and transfected and nontransfected HEK293t cells, these were fixated with paraformaldehyde 4% with glutaraldehyde 0.2% for 15 minutes on ice for DRGs or 10 min on ice for HEK293t cells, washed with PBS 1×, and incubated with permeabilization solution (0.2% saponin, 0.1% fish soluble gelatin in PBS 1×) for 60 minutes at 25°C. Cells were then incubated with the respective primary antibodies diluted in blocking solution (0.1% fish soluble gelatin in PBS 1×) overnight at 4°C. After that, cells were washed with PBS 1× three times for 5 minutes, incubated with the corresponding secondary antibodies diluted in blocking solution for 1 hour at room temperature, and washed with PBS 1× three times for 5 minutes. To stain the nuclei, DRGs and HEK293t cells were incubated with DAPI 1:1000 for 2 minutes at room temperature, washed with PBS 1× three times for 5 minutes, and mounted in 1:6 PBS:Glycerol. All substances were obtained from Sigma‐Aldrich. Rabbit anti‐mTRPA1 (ARP35205_P050, Aviva Systems, CA, USA) at 1:500 was used as primary antibody and donkey anti‐rabbit Dy650 (Abcam, Cambridge, UK) at 1:600 as secondary antibody. Fluorescent images were acquired with a confocal microscope with laser excitation wavelengths of 405 and 640 nm (Nikon Eclipse Ti A1, Tokyo, Japan) or with an epifluorescence microscope with LED‐based excitation at 405, 490, and 650 nm (Olympus IX71). Image analysis was performed with ImageJ.

### 4.10 Statistical methods

#### 4.10.1 Pharmacological stimulation of single cells

Cardiomyocytes or DRG neurons were manually selected by a calcium increase in response to depolarization by KCl 60 mmol L^‐1^. Additionally, cardiomyocytes with substantial spontaneous activity were thought to be in poor condition and were removed if the skewness of the distribution of the fluorescence signal in the period before the first chemical application was outside a range of −0.5 to 0.5. A quadratic polynomial, chosen to account for the curvature of the traces, was fitted to the data using the least squares method to the period before agonist application. In DRGs, basal calcium was stable and a period of 5 seconds before stimulation was used. For the periods of substance application the regression lines were extrapolated to provide an expected development without stimulation. Area under the curve (AUC) values were calculated for the period of agonist stimulation according to the trapezoidal rule.

#### 4.10.2 Electrically paced primary mouse cardiomyocytes

The analysis was limited to cardiomyocytes regularly responding to electrical pacing. The following calculations were performed for each cell: to account for the curvature of traces, a quadratic polynomial was fitted to the last 5 data points (acquired every 0.1 seconds) before electrical stimulation #2‐14, using the least squares method. The regression line was interpolated or extrapolated and provides a robust estimate of the expected values without stimulation. AUC values were calculated for a period of 1 second after stimulation and for the 30 seconds KCl application period. The statistical analysis tested whether the electrically stimulated AUCs differed between compounds (Figure S3). The first two AUCs after the start of the application were omitted from the analysis to consider the binding kinetic of the substances. The adjustment AUCs labelled in the figure were each used as covariate to adjust the estimated treatment differences for differences arising from the cell's condition. The following mixed model approach was applied. The compound type, the animal, and the control AUCs were used as fixed factors; each cardiomyocyte was used as the level of a random factor. Based on the Akaike information criterion, a diagonal covariance structure was chosen. *P* values and confidence intervals were adjusted for four comparisons with the control using Bonferroni's method. Approximate normal distribution was checked graphically by plotting the predicted values versus the residuals.

#### 4.10.3 Pharmacological stimulation of HL‐1 cells

This cardiomyocyte cell line exhibits spontaneous beating and therefore calcium waves. The frequency and amplitudes of calcium peaks before (0‐180 seconds) and after (210‐390 seconds) a 30 seconds agonist application were compared. To identify peaks and get their amplitudes, the findpeaks function of MATLAB version R2019a was used with settings of ‘MinPeakDistance’ = 1, ‘MinPeakProminence’ = 0.1, ‘MinPeakHeight’ = 0.75 × median amplitude of the signal. To find out whether amplitude or frequency changed before to after agonist application, a mixed model was used. Statistical analysis was performed using IBM SPSS statistics 25‐27 (Armonk, NY, USA). *P* values ≤.05 were considered statistically significant. Graphs were generated using GraphPad Prism 8 (Graphpad Software, Inc, San Diego, CA, USA), Sigmaplot 14 (Systat Software Gmbh, Erkrath, Germany), or R with RStudio (RStudio PBC, Boston, MA) and arranged in CorelDraw 17‐22 (Corel Corporation, Ottawa, Canada).

## CONFLICT OF INTEREST

There is no conflict of interest.

## Supporting information

Fig S1

Fig S2

Fig S3

Fig S4

Fig S5

Table S1

## Data Availability

The data that support the findings of this study are available from the corresponding author upon reasonable request.
