# Supplementary figures and images for: No functional TRPA1 in cardiomyocytes
Source: Acta Physiol (Oxf). 2021 May 4;232(4):e13659. doi: 10.1111/apha.13659 (PMC11478933; doi:10.1111/apha.13659)

F340/F385

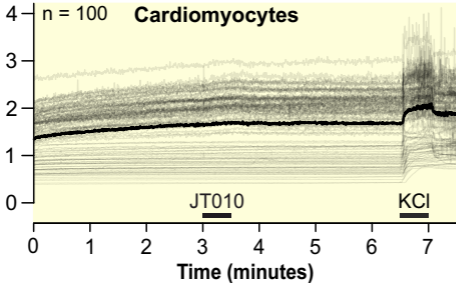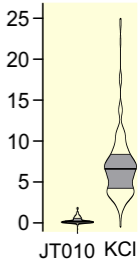

Supplement: Supplementary file 1 — Fig S1 [file APHA-232-e13659-s001.pdf]

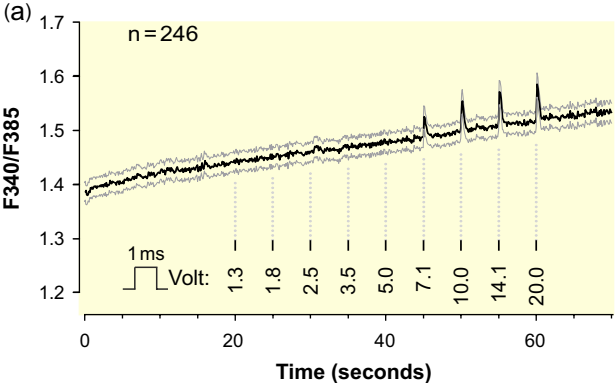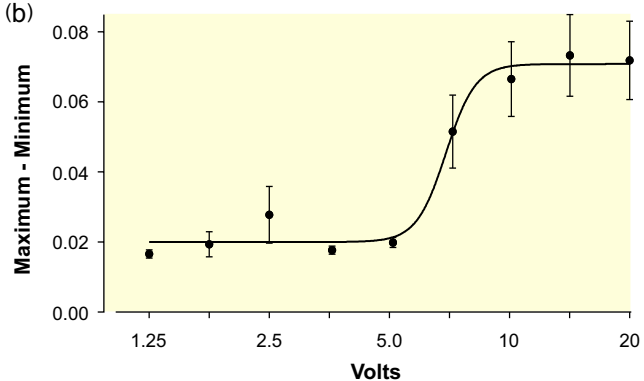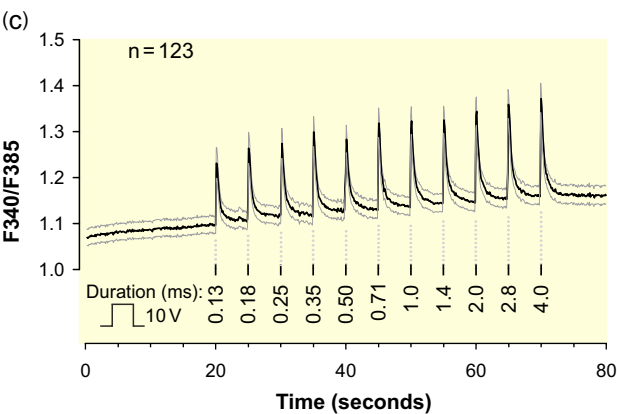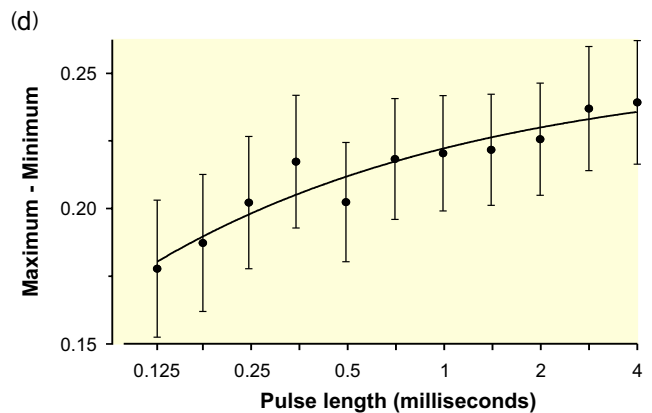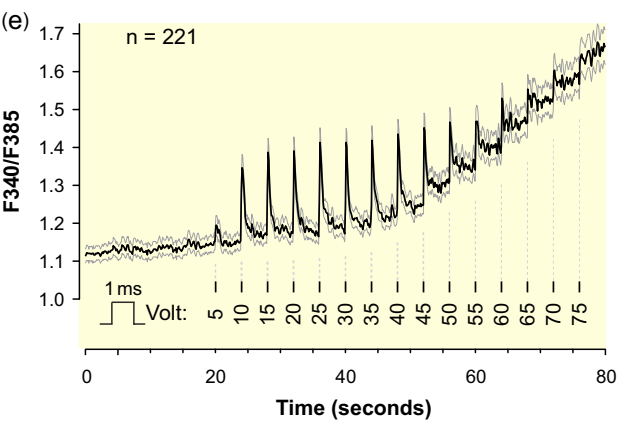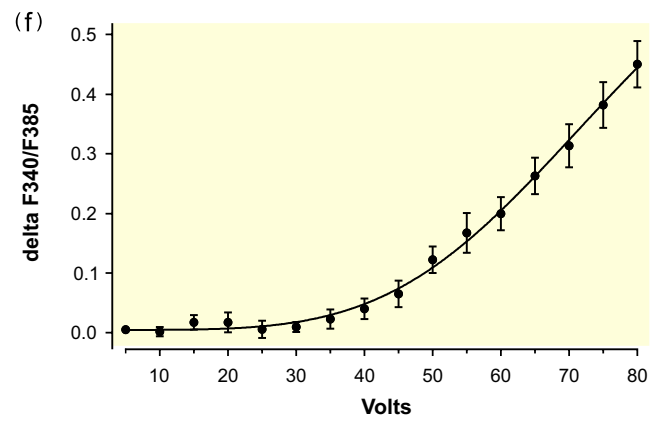

Supplement: Supplementary file 2 — Fig S2 [file APHA-232-e13659-s005.pdf]

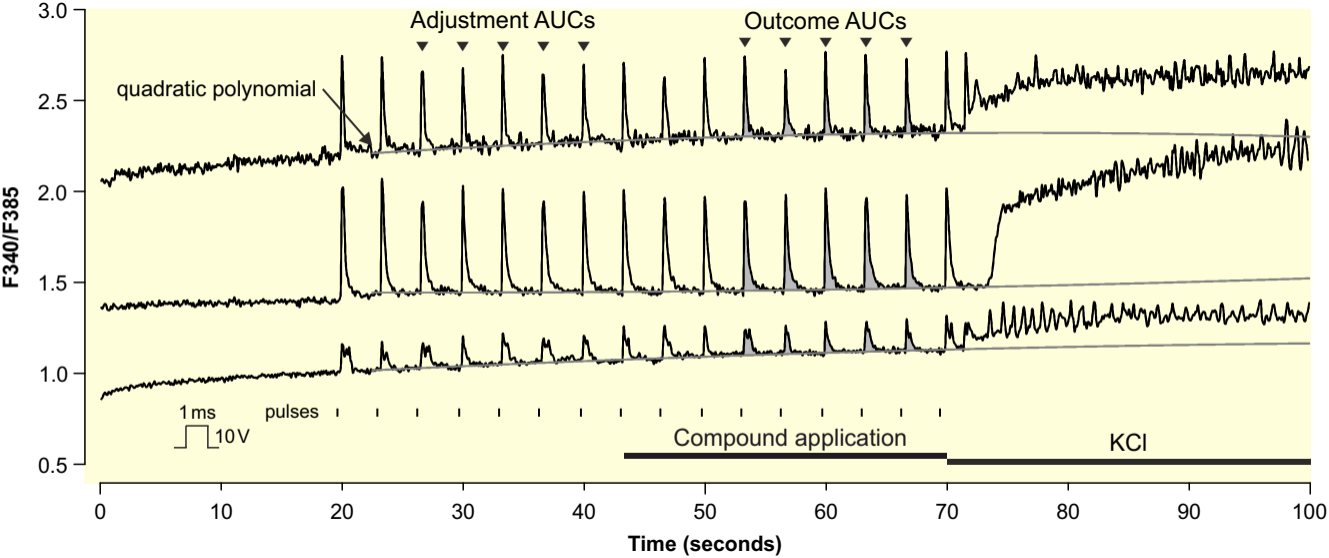

Supplement: Supplementary file 3 — Fig S3 [file APHA-232-e13659-s006.pdf]

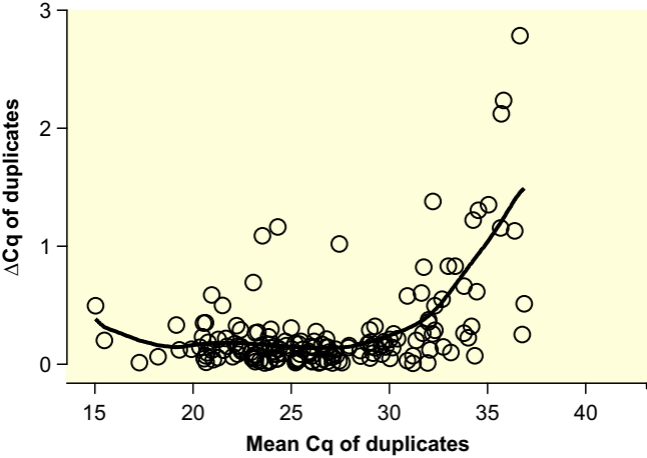

Supplement: Supplementary file 4 — Fig S4 [file APHA-232-e13659-s004.pdf]

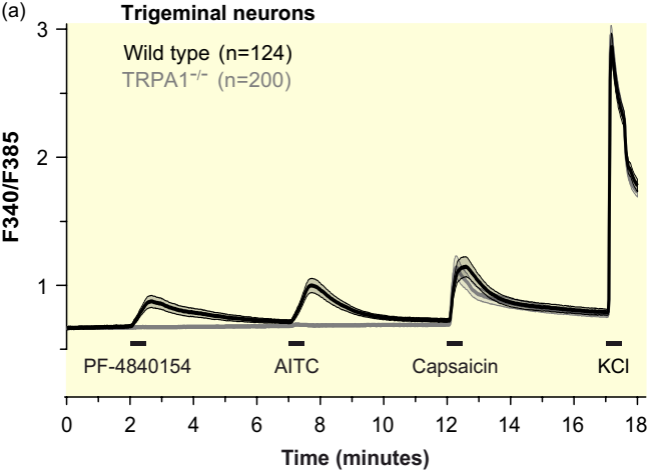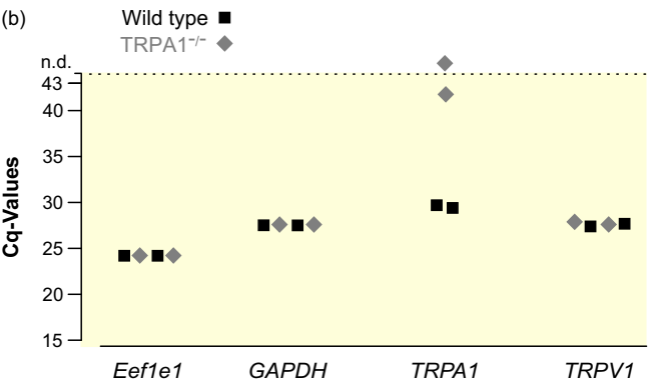

Supplement: Supplementary file 5 — Fig S5 [file APHA-232-e13659-s003.pdf]
