# Supplementary material for: No functional TRPA1 in cardiomyocytes
Source: Acta Physiol (Oxf). 2021 May 4;232(4):e13659. doi: 10.1111/apha.13659 (PMC11478933; doi:10.1111/apha.13659)
Supplement: Supplementary file 6 — Table S1 [file APHA-232-e13659-s002.docx]

Supplementary Table 1: Genes of interest and corresponding primer sequences

| Gene symbol | Gene name | Species | Sequences (5'to3') |
| --- | --- | --- | --- |
| GAPDH | Glyceraldehyde 3-phosphate dehydrogenase | *R. norvegicus/M. musculus* | F: GACATGCCGCCTGGAGAAAC |
|  |  |  | R: AGCCCAGGATGCCCTTTAGT |
| Eef1e1 | Eukaryotic translation elongation factor 1 epsilon 1 | *M. musculus* | F: GACAAAACCAGCGAGACACA |
|  |  |  | R: GACATAACATCACCCTGGCG |
|  |  | *R. norvegicus* | F: GGCTCCACCGCTTTATAGTTG |
|  |  |  | R: AGTGGGAGTTGGCATAGAGT |
| Actin B | Beta-actin | *H. sapiens* | F: GCACAGAGCCTCGCCTTTG |
|  |  |  | R: ATCCATGGTGAGCTGGCG |
| IPO8 | Importin-8 | *H. sapiens* | F: ATAGGTCCAGGGTTGTGAGG |
|  |  |  | R: TCAGTGCAAAGGAAGGGGAA |
| TRPA1 | Transient receptor potential cation channel subfamily A member 1 | *M. musculus* | F: TTGGATATTGCAAAGAAGTGATCC |
|  |  |  | R: GAGGAACAAGGGCAACACGA |
|  |  | *R. norvegicus* | F: GCAGCATTTTCAGGTGCCAA |
|  |  |  | R: CGCTGTCCAGGCACATCTTA |
|  |  | *H. sapiens* | F: TATTCCCTCACTACCCCCAGG |
|  |  |  | R: CACAAGGACACATACATAGCCA |
| TRPV1 | Transient receptor potential cation channel subfamily V member 1 | *M. musculus* | F: CATGCTCATTGCTCTCATGG |
|  |  |  | R: AGGCCTTCCTCATGCACTTC |
|  |  | *R. norvegicus* | F: GAATGACACCATCGCTCTGC |
|  |  |  | R: AAGAGGGTCACCAGCGTCAT |
|  |  | *H. sapiens* | F: TCAACAAGATCGCACAGGAG |
|  |  |  | R: GCCTGAAACTCTGCTTGACC |
